# Economic effects of foot and mouth disease outbreaks along the cattle marketing chain in Uganda

**DOI:** 10.14202/vetworld.2016.544-553

**Published:** 2016-06-02

**Authors:** Sylvia Angubua Baluka

**Affiliations:** Department of Biosecurity, Ecosystem & Veterinary Public Health, College of Veterinary Medicine, Animal Resources & Biosecurity, Makerere University, Kampala, Uganda

**Keywords:** chain, cost, economics, financial losses, market, outbreak

## Abstract

**Aim:**

Disease outbreaks increase the cost of animal production; reduce milk and beef yield, cattle sales, farmers’ incomes, and enterprise profitability. The study assessed the economic effects of foot and mouth disease (FMD) outbreaks along the cattle marketing chain in selected study districts in Uganda.

**Materials and Methods:**

The study combined qualitative and quantitative study designs. Respondents were selected proportionally using simple random sampling from the sampling frame comprising of 224, 173, 291, and 185 farmers for Nakasongola, Nakaseke, Isingiro, and Rakai, respectively. Key informants were selected purposively. Data analysis combined descriptive, modeling, and regression analysis. Data on the socio-economic characteristics and how they influenced FMD outbreaks, cattle markets revenue losses, and the economic cost of the outbreaks were analyzed using descriptive measures including percentages, means, and frequencies.

**Results:**

Farmers with small and medium herds incurred higher control costs, whereas large herds experienced the highest milk losses. Total income earned by the actors per month at the processing level reduced by 23%. In Isingiro, bulls and cows were salvage sold at 83% and 88% less market value, i.e., a loss of $196.1 and $1,552.9 in small and medium herds, respectively.

**Conclusion:**

All actors along the cattle marketing chain incur losses during FMD outbreaks, but smallholder farmers are most affected. Control and prevention of FMD should remain the responsibility of the government if Uganda is to achieve a disease-free status that is a prerequisite for free movement and operation of cattle markets throughout the year which will boost cattle marketing.

## Introduction

Uganda’s well-being is inextricably tied to livestock with 70% of households owning cattle, goats, sheep, pigs, or chicken. About 22% and 60% of households nationally and in the cattle corridor, respectively, derive their livelihood from livestock [1,2]. Animal diseases are a major constraint to livestock production and trade in Uganda [3]. The presence of infectious diseases such as foot and mouth disease (FMD) limits Uganda’s ability to access major export markets, and her performance in the global export trade in livestock and livestock products is negligible due to FMD [4]. Frequent FMD outbreaks and subsequent bans on cattle and cattle products movement to and from the affected areas during quarantine period cripples cattle marketing [3,5]. Furthermore, livestock diseases impose heavy costs on farmers and reduce incentives to invest in higher yielding cross breeds or exotic animals that are more susceptible to tropical diseases [2].

FMD causes serious production losses, is highly contagious, transmitted through multiple routes and hosts which makes it one of the most important diseases affecting trade in livestock [6-8]. Animal diseases affect the amount as well as the timing and certainty of income from livestock enterprises and undermine the livestock sector potential, compromise food security, affect livelihoods by reducing milk yield and body weight while increasing losses due to disease control costs and mortalities [9-11]. These diseases reduce the level of sales for cattle and cattle products [12], perceived or actual output quality, input utilization efficiency [13], necessitate international trade restrictions thus reducing the benefits derived by mankind from livestock farming [14,15], and cause losses of up to 30% of the annual livestock output in developing countries [16].

Despite 75% of the world’s cattle living in low- and middle-income countries, livestock product exports from these countries account for less than 15% of the global value [17] with exception of Brazil that is ranked among the largest beef producers and exporters globally [18]. This paradox is partly explained by the presence of transboundary animal diseases (TADs) such as FMD that restrict livestock trade [9,19] and often precede uncontrolled livestock movements typical of grazing systems in these countries [20,21]. Controlling production costs are important in modern livestock farming. Improving animal health and fertility can play a major role in achieving efficient and economically rewarding production [22,23]. Policy makers and livestock industry stakeholders need means of assessing a disease’s economic impacts when evaluating prevention and mitigation measures [24]. This study assessed the economic costs incurred by the various actors along the cattle marketing chain during FMD outbreaks in selected study districts.

## Materials and Methods

### Ethical approval

The research proposal and data collection instruments that led to this manuscript were vetted and approved by the College of Veterinary Medicine, Animal Resources and Biosecurity, Makerere University Research Ethics Committee.

### Study design

The study combined qualitative and quantitative study designs. Desk review of disease outbreak reports in Ministry of Agriculture Animal Industry and Fisheries archives was done. The study was phased into reconnaissance visit that involved site selection and participatory discussions, semi-structured interviews with key stakeholders (extension staff, policy makers, and local leaders), survey using a structured questionnaire and case studies. The qualitative design employed focus group discussions (FGDs) and key informant interviews (KIs).

### The study population, study design, and sample size determination

Livestock markets from the sub-counties most affected by the previous FMD outbreaks were identified, and they provided a nucleus for selecting study sites. Two livestock markets per district were selected for the study, which involved three stratified populations: Farmers, livestock traders/transporters, and processors. The herd was the unit of study at the farmers’ level, and it was most appropriate given the epidemiology and morbidity of FMD within infected herds.

The catchment areas (parishes) that supplied livestock to the selected livestock markets were identified, and sampling frames were constructed to include farmers from all the listed parishes with the help of extension veterinarians and farmers’ association leaders. Sample sizes from each stratum were obtained using proportional sampling. The sampling frames comprised 224, 173, 291, and 185 farmers for Nakasongola, Nakaseke, Isingiro, and Rakai districts, respectively. The study farms from each district were selected from the sampling frame using simple random sampling. Veterinary extension staff, local leaders, and farmers for the KIs and FGDs were selected purposively. Traders were considered as an aggregate rather than by district since they operate in several districts and a total of 45 cattle traders were interviewed.

FMD case studies were done in 17 herds that had experienced FMD outbreaks in Mbaare, Isingiro; 3, 9, and 5 herds were selected to represent small, medium, and large herds. Small, medium, and large herds refer to herd sizes of 10-50, 51-150, and 151-350 heads of cattle, respectively.

### The main questions asked during FGDs

The main questions asked during FGDs with cattle farmers addressed the major livelihood activities, type of cattle production system used, cattle marketing channels and mode of transport used, and impact of FMD on cattle prices and household incomes.

The main questions asked during the FGDs with cattle traders included the following: How often do you buy cattle for sale? Where do you buy cattle from and where do you resale them? Where do you obtain information on markets and cattle prices from? Have you ever sold cattle outside Uganda’s borders, i.e., to other neighboring countries? At what price did you buy/sell cattle before, during and after the FMD outbreak? How many animals did you buy/sell cattle before, during, and after the FMD outbreak? Were you able to get alternative suppliers of animals during FMD outbreak? Which extra costs did you incur during the FMD outbreak?

The main questions asked during the FGDs with cattle processors included; how often do you buy/slaughter cattle? Where do you normally buy your slaughter cattle from? How many people do you employ in your cattle slaughter/butchery business? Where do you obtain information on cattle markets and prices from? How do you transport your purchased cattle from the cattle markets to the holding farm/slaughter place? How was your cattle slaughter business affected during the last FMD outbreak?

The main questions asked during the key KIs with Veterinary Extension staff at the districts, i.e. DVOs included how long they had served in the current employment in the study district, the major constraints/challenges affecting livestock farming in the district, whether they had experienced outbreaks in their district in the period under study.

The main questions asked during the KIs with policy makers and regulators from the ministry included; the laws and regulations regarding prevention and control of TADs, number of outbreaks and cases reported in the area in the period under study, policies, and institutional arrangements for control of FMD.

### Structured questionnaire interviews

Structured questionnaire interviewing was the most appropriate technique for gathering data to quantify the economic effects of FMD at the production and marketing levels because it gives the researcher control over the process, achieves high response rates, and ensures complete information.

### The main questions asked during the structured questionnaire interviews with farmers

The farmers’ structured questionnaire survey captured information on household demographics such as sex, age, education level, and experience in years in livestock rearing for the household head, reasons for keeping cattle, household assets and income sources, land ownership tenure or user rights, sources of market information, major channels used for selling cattle, major mode of transportation of cattle to the market, major channels for sales and purchases of cattle, source of breeding stock, vaccination schedules against FMD, husbandry practices in regards to feeding and watering, and major inputs into cattle production, costs associated with FMD control and treatment, constraints to cattle production and marketing and overall impact of FMD on farmers.

### The main questions asked during the structured questionnaire interviews with cattle traders

The traders’ structured questionnaire survey captured information on demographics such as sex, age, educational level, years in cattle trading business, major livelihood activity and income source, cattle commodity traded, sources, and amount of capital, scale of operations, percentage contribution of cattle trade toward their household income, number of workers employed in their cattle trading business, how much they pay the workers per month or in kind, availability of cattle holding grounds in their area of operation, mode of transportation of cattle, whether they use brokers or middlemen, quality attributes sought for when purchasing cattle, percentage of cattle purchases by different channels, constraints in livestock marketing, access to cattle marketing facilities, source of marketing information, volume of cattle sold per week, impact of FMD on cattle and beef prices during outbreaks, major opportunities and constraints in cattle trading.

The main questions asked during the structured questionnaire interviews with cattle processors/slaughter facility operators captured information on major livelihood activity, years in cattle processing business, whether they have received any specialized training in cattle processing, major income sources and percentage contribution per month, number of workers employed, how much they pay the workers in cash or kind per month, whether they belong to any association or cooperative for processors, legal status of their business, quality attributes sought for when purchasing beef, common terms of payment in their trade, constraints faced in their business, source of market information, how the FMD outbreak affected cattle and beef prices. Data on slaughter capacity, number of livestock species slaughtered in the facility, number of days the slaughter facility operates and changes in slaughter volumes during outbreaks were also captured.

### Determination of market revenue earned from livestock markets

Market revenue analysis considered bad and good cattle marketing seasons. The bad season falls within the dry season when cattle move long distances in search for pastures and water, they lose weight and are generally in poor body condition, the total cattle turnover in markets and prices are low. The good season occurs during the rainy season when cattle have enough pasture and water; they are in good body condition, total cattle turnover in markets and prices are high. The good season also includes festive seasons such as Christmas, Easter, and Eid. The total annual market revenue losses were assumed equal to total income earned from cattle during good season months + total income earned from cattle sales during the bad season months.

Where by:

Annual total income earned from cattle during good season months=number of months in a year, in which cattle were sold during good season*price levied per head of cattle*average number of cattle sold per market day. Annual total income earned from cattle during bad season months=number of months in the year, in which cattle are sold during bad season*price levied per head of cattle*average number of cattle sold per market day. Estimation of cattle market revenue losses was based on the assumption that if the outbreak lasted 14 months, the quarantine period was 6 months; 2 months fell within the good season and 4 months fell within the bad season. Moreover, if an outbreak lasted for 6 months or more then the quarantine period lasted for 1-year or longer. The cattle prices levied per head of cattle were made comparable to price values charged during 2010 by compounding the prices charged in the previous years (2006-2009) using the formula:

P=P_i (1+r)_^n^

Where,

P= Discounted price in previous year earlier than 2010

P_i_=Price during previous year earlier than 2010

n=number of years before 2010

r=discounting rate of 7%.

The market losses in the two district categories (inland and border districts) and between districts within the same district category were estimated. The significant difference in revenue was determined using a Chi-square test. The market revenues were determined under the following scenarios:

- Revenue earned when there was no FMD outbreak
- Revenue earned during FMD outbreaks
- Income lost during FMD outbreaks.

### Determination of household incomes

Household incomes were determined in a four step process:

- Identifying and listing all the major inputs into the cattle enterprises. These included farm infrastructures such as fence maintenance, hired labor, water, tick control costs, vaccination costs, treatment costs, mineral salts, and supplements.
- Obtaining the unit cost of each input and then determining the average annual household expenditures on the various inputs into a given cattle enterprise.
- Identifying and listing all the various outputs from a given cattle enterprise. The major outputs included milk, manure (cow dung that is sold in most study districts apart from Nakasongola), and sale of live cattle.
- The last step involved quantifying how much of the outputs were produced per household per week, month and then computed the average household income earned annually from the cattle enterprise.

### Gross margin analysis (GMA)

GMA was performed by a spreadsheet model developed using Microsoft Excel Version 5.0, Microsoft Corporation, USA, on households when there were no FMD outbreaks using [25] procedure. GM was taken as the gross income of an enterprise less variable costs. Total livestock income or output was taken as a sum of income from the sale of live animals, milk, and manure. Draft power was not important in the study areas and was, therefore, excluded from the analysis. GM can be represented by the equation: GM=∑ (y_i_ × py_i_) − ∑ (X_i_ × PX_i_)

Where,

∑ (yi × pyi) = Gross income,

Y_i_ = Quantities of products,

Py_i_ = Respective prices of the products,

∑ (X_i_ × PX_i_) = Total variable costs,

X_i_ = Quantities of costs,

Px_i_ = Respective prices of variable inputs.

### Determination of economic cost of FMD outbreaks in the case study herds

The FMD economic cost comprised of the control costs (vaccination and treatment of secondary infections in affected cattle herds), losses due to weight loss, abortion, losses in milk production, reduced manure production, salvage sales during the outbreak and mortality. The economic cost of FMD outbreaks in the case study herds were calculated as follows:

Milk yield losses for rainy and dry seasons = Average herd size × % lactating cows × milk yield × % milk reduction.

Herd-specific abortions losses = Average herd size × % Cows ×Average number of calves per year × % calf reduction.

Age-specific mortality losses for bulls, cows, heifers, and calves=Age-specific prices × Herd size × % Composition × % dead.

Age-specific weight losses for bulls, cows, heifers, and calves = Herd size × % Bulls × Average weight of bulls × % weight reduction.

Treatment costs for FMD=Average herd size × % infected × Average cost of treatment per head.

Vaccination costs for FMD = Average herd size × Average cost of vaccination per head × Number of vaccinations per year.

Age-specific salvage sale losses were calculated by considering cattle prices before, during, and after the last FMD outbreak.

Age-specific salvage sale losses for bulls, cows, heifers, and calves (female and male) = Age-specific prices × Herd size × % Composition × % Price reduction.

### Data analysis

The study used qualitative data, which were captured using FGDs, semi-structured questionnaire; KIs and quantitative data captured using the structured questionnaire. Qualitative data were analyzed by summarizing content according to the major themes. Quantitative data were entered using MS Excel and analyzed using STATA. Data from case studies were analyzed using regression analysis. Data analysis combined descriptive and regression analysis with inferential statistics to compare market revenue losses and economic costs during FMD outbreaks.

## Results

### Financial analysis of cattle enterprises in the study districts

The major inputs into the cattle enterprises included labor, treatment or veterinary drugs, acaricides (tick control chemicals), mineral salts, vaccination, fencing, and farm maintenance. While the major outputs from cattle enterprises were milk, live cattle sales, and manure. The average household daily milk production including the amount consumed, sold and given to herdsmen as a form of payment for their labor during the rainy and dry seasons and the prices per study district were as shown in Table-1. In all the study districts, milk yield was significantly different between the dry and wet seasons. The variance was more prominent in Nakasongola compared to the rest of the study districts. Prices increased and fell in the dry and wet seasons, respectively, in all the districts, but the variance was more prominent in Rakai where the prices increased by nearly 50% (Table-1).

**Table-1:** Average household daily milk production, consumption, and sales; and prices during the wet and dry seasons in the study districts.

Item	Season	Nakasongola	Nakaseke	Isingiro	Rakai
Daily milk yield (liters)	Wet	17.5	51.6	23.9	12.0
	Dry	6.0	25.5	11.3	6.6
Daily milk consumption	Wet	4.5	9.5	12.6	9.2
	Dry	2.3	8.6	7.5	5.0
Daily milk sales	Wet	13.0	42.0	11.4	3.0
	Dry	3.9	16.8	3.7	1.7
Milk payment to herdsmen in-kind	Wet	3.0	4.7	5.3	2.5
	Dry	1.4	2.1	2.9	1.0
Prices/liter (UGX)	Wet	326.8	270.0	320.0	492.9
	Dry	420.1	380.3	421.5	632.1

Nakaseke earned higher revenues from milk annually compared to the rest of the study districts. Ironically, all the study districts earned more revenues from milk during the dry season than in the wet season (Table-2).

**Table-2:** Average annual total revenue from milk in US dollars per household in the study districts.

Outputs	Season	Nakasongola	Nakaseke	Isingiro	Rakai
Milk sales	Wet	450	1201	386	201
	Dry	174	677	165	114
Ghee sales		548	98	130	193
Home consumption	Wet	156	272	427	480
	Dry	102	346	335	335
Milk payment to herdsmen in-kind	Wet	104	209	180	131
	Dry	62	85	129	67
Total output	Wet	1258	1780	1123	1004
	Dry	338	1107	629	515
Average total output		798	1443	876	760

During the normal marketing periods (without outbreaks), bulls and cows fetched higher prices than other cattle types in all the study districts. Notably, the prices for bulls and cows are much higher in Nakaseke than in the other study districts (Table-3). Farmers in all the four study districts mainly sell cows (culled animals) as compared to the other age groups, which explains the generally low off-take rate (Table-4).

**Table-3:** Normal average age-specific prices of cattle (US dollars) in the study districts.

Category	Nakasongola	Nakaseke	Isingiro	Rakai
Female calves	77	66	52	121
Male calves	77	66	52	59
Steers	132	206	59	127
Heifers	162	235	147	127
Cows	265	309	221	217
Bulls	438	577	309	309

**Table-4:** Percentage age-specific off-take sale composition per district between January 2010 and January 2011 as revealed by sub-county livestock markets records.

Category	Rakai	Isingiro	Nakasongola	Nakaseke
Female calves	14.8	10.8	8.4	2.4
Male calves	21.6	19.0	5.3	6.2
Steers	3.4	9.0	9.9	4.1
Heifers	23.5	12.5	23.2	9.8
Cows	35.7	46.0	48.2	72.8
Bulls	0.9	2.6	5.0	4.7

On average, households in Nakaseke earned more revenue from cattle sales than households in other study districts (Table-5). Nakaseke had a higher off-take rate and earned more income from cattle sales compared to the rest of the study districts (Table-6).

**Table-5:** Total cattle populations, number of cattle sold, revenue earned (US dollars) from cattle sales, and number of households that sold cattle per district.

District	Total cattle population	Total number of cattle sold	Total revenue earned from cattle sales	Number of households that sold cattle per district
Isingiro	396,700	918	136,618	330
Rakai	279,394	582	84,332	205
Nakasongola	222,185	604	127,143	242
Nakaseke	160,737	1717	496,088	246

**Table-6:** The percentage of the cattle herd sold and average annual household income in US dollars earned from cattle sales per district.

District	% of cattle herds sold	Average annual household income from cattle sales
Isingiro	0.23	414
Rakai	0.28	411
Nakasongola	0.27	525
Nakaseke	1.07	2017

Milk yield loss during FMD outbreaks affected households in all the study districts, and it resulted into loss of income from milk and live cattle sales (Table-7). There was a significant difference in income lost due to a decrease in milk and live cattle sales. The loss of income due to milk loss and live cattle sales was more significant in Nakasongola compared to Rakai (Table-8).

**Table-7:** Effects of milk yield loss (percentage) on households during FMD outbreaks.

Effects of milk yield losses on the household	Nakasongola	Nakaseke	Isingiro	Rakai
Starvation due to loss of milk for food	26.8	8.7	30.7	33.9
Loss of income from milk and live cattle sales	69.5	58.4	48.6	25.6
Increased household expenditure on drugs, vaccines, and labor	14.1	31.9	20.7	40.5

FMD=Foot and mouth disease

**Table-8:** Loss of income due to milk loss and live cattle sales.

Districts compared	χ^2^ value	Level of significance difference
Nakasongola versus Rakai	38.4	p<0.001
Nakasongola versus Isingiro	5.6	p<0.01
Nakaseke versus Isingiro	2.7	p>0.05
Rakai versus Nakaseke	22.5	p<0.01
Rakai versus Isingiro	11.7	p<0.01

The most significant difference between farmers’ expenditure on drugs, vaccines, and labor during FMD outbreaks was observed in Nakasongola as opposed to Rakai (Table-9). Another major effect of FMD on the farmers during outbreaks was via abortion. Foot and mouth disease outbreaks were associated with abortions in all the study districts; (84.7%) for Isingiro, 100% for Nakasongola and Nakaseke, and 76.7% for Rakai.

**Table-9:** Household expenditure on drugs, vaccines, and labor during FMD outbreaks.

Districts compared	χ^2^ value	Level of significance difference
Nakasongola versus Nakaseke	8.14	p<0.01
Nakasongola versus Isingiro	1.36	p>0.05
Nakasongola versus Rakai	17	p<0.001
Rakai versus Nakaseke	1.8	p>0.05

FMD=Foot and mouth disease

FMD control cost per head of cattle was highest in the small herds ($19), more than thrice and eight times for medium and large herds, respectively. Mortality losses in small and medium case study herds were $429 and $214, respectively, and there was no mortality in large herds. The salvage losses in small and medium case study herds were $143 and $113, respectively, and there were no salvage losses in large herds, i.e. large herds did not sell any cattle at salvage prices during outbreaks and quarantine (Table-10).

**Table-10:** FMD control costs in case study herds in Isingiro district.

Control item	Herd category	Small	Medium	Large
Treatment	Bulls	57	29	0
	Cows	86	357	143
	Heifers	29	29	214
	Steers	0	0	0
	Calves	43	43	114
	Total	214	457	471
Vaccination costs		4.3	23	74
Veterinary costs		6	6	6
Total FMD control costs		224	487	551
FMD control cost per head of cattle		19	6	2

FMD=Foot and mouth disease

The economic loss due to the reduction of milk production during FMD outbreak and losses due to the absence of milk sales during the quarantine period were as shown in Table-11. Average milk yield per cow was 0.9±0.45 and 2.1±0.3 during dry and wet season, respectively, with each liter of milk costing $0.12 and $0.09, respectively. During FMD outbreaks, there was 42% drop in milk yield in infected cattle over a period of 12 weeks. 12% of the milk produced which is usually sold was not sold during this period. During FMD quarantine periods, milk production gradually resumes in recovered cattle; however, only 12% of the milk was sold (Table-11).

**Table-11:** Milk production losses in US dollars due to lack of milk sales during FMD outbreaks in case study herds.

Herd size category	Loss due to reduction of milk production during FMD outbreak	Loss due to absence of sales during FMD outbreak	Milk sale loss caused due to FMD quarantine	Total milk loss due to FMD
Small herds	42	11	213	267
Medium herds	144	43	441	629
Large herds	613	183	1873	2670
Average	266	80	814	1160
% composition of type of milk loss to total milk economic loss	23	6.9	70.1	

FMD=Foot and mouth disease

Mortality loss, salvage sale loss, and milk loss accounted for the greatest percentage of the total economic cost due to FMD in small, medium, and large herds, respectively, in case study herds in Isingiro (Table-12).

**Table-12:** The percentage of each economic cost to total FMD cost in Isingiro district.

Economic loss		Small		Medium		Large
Mortality loss		40		9		0
Salvage sale loss		13		46		0
Milk loss		25		26		83
Treatment		20		19		15
Vaccination		0		1		2
Veterinary costs		1		0		0

FMD=Foot and mouth disease

Large herds spent more on vaccination than the small and medium herds (Table-13).

**Table-13:** Average vaccination costs in US dollars against FMD for all herd categories.

Age category		Small		Medium		Large
Bulls		1		2		6
Cows		5		12		25
Heifers		3		7		6
Steers		0.3		3		1.2
Calves		2		7		5.3
Total		10		30		43

FMD=Foot and mouth disease

The potential total number of cattle that can be sold in the study markets annually and the representative total off-take per district when there was no FMD outbreak were as shown in Table-14.

**Table-14:** Annual potential volume of cattle that can be sold per market and percentage off-take from two markets per district.

District	Market	Number of cattle sold yearly	% off-take of the total district herd
Nakasongola Nakaseke	Wabinyoyi	4500	10.7
	Nakitoma	19200	
	Subtotal	23700	
Rakai	Ngoma	19200	19.4
	Kinyogoga	12000	
	Subtotal	31200	
	Kibanda	7200	5.2
	Kakuuto	7,320	
	Subtotal	14520	
Isingiro	Bugango	8880	3.6
	Rwenseke	5400	
	Subtotal	14280	

The inland districts (Nakaseke and Nakasongola) had higher off-take rates than the international border districts (Rakai and Isingiro) as shown in Table-14. The inland districts would earn very highly significant income (p<0.001); Nakaseke and Nakasongola had the potential to earn 38.6% and 34.1% of the grand total earned by all study districts, respectively, as compared to districts along international borders. The total cattle markets revenue loss during FMD outbreaks from 2006 to 2010 amounted to $340543. The international border districts lost less income (p<0.001, $122514, 47.2%) of the grand total than the inland districts ($137371, 52.9%) during FMD outbreaks in the 5-year period under study.

The total income earned by processing actors per head of cattle during the outbreak and non-outbreak periods in Nakasongola district is presented in Table-15. The total income earned by the actors per month at the processing level reduced by 23% during outbreaks (Table-15).

**Table-15:** Total income earned (US dollars) by processing actors per head of cattle during outbreaks and non-outbreak periods in Nakasongola district.

Actor	Income earned weekly without outbreak	Income earned monthly without outbreak	Income earned weekly during outbreak	Income earned monthly during outbreak
Weighing scale owner	30	120	1	24
Local government	30	120	1	24
Veterinary inspectors	30	120	1	24
Public health inspector	30	120	1	24
Slaughter man/moslem	30	120	1	24
Other operational costs	86	343	1	23
Butchery workers	51	206	0.4	41
Processors	2700	10800	26	2571
Total income	2987	11949	689	2755

## Discussion

Milk yield loss during FMD outbreaks affected farming households in all the study districts, and it resulted into loss of income due to a decrease in milk sales. However, FMD outbreaks affected households in the study districts differently. For instance, loss of income due to loss of milk and live cattle sales was more significant in Nakasongola than in Rakai.

The economic cost per head of cattle was highest in the small herds followed by the medium herds and was lowest in the large herds. The greatest percentage of the economic cost due to FMD arose from milk losses in large herds compared to the economic costs in small and medium herds that were mainly due to market blockage caused by continued persistence of the disease in the affected cattle farming areas.

Vaccination comprised a major control cost although FMD remains endemic in the study area. An optimal strategy for FMD control in Uganda is yet to emerge, and it will only be possible if the unique circumstances such as production systems, livestock movements to the market are considered as recommended [26]. Thus, FMD disproportionately affects the smallholders and probably the poorer households who may not afford the cost of prevention and control of the disease. In calf cows aborted as a result of FMD, abortions affect the herd structure and undermine the farmers’ economic objective of one calf per cow per year [27]. FMD related age-specific mortality as perceived by farmers was generally low, and calves were most affected which agrees with [20]. FMD outbreaks had food security effects on the farmers by causing starvation which is in agreement with previous studies [28,29].

Processors experienced physical loss of meat products and negative demand shifts which greatly reduced the price of beef and profitability, and these findings are in tandem with [23]. In Nakaseke, under normal circumstances processors earned net monthly income of about $14 after paying off all the operational costs, but this income was lost during the outbreaks. Processors found themselves out of employment, i.e. slaughters reduced from 5 heads of cattle before FMD outbreak to 1/day.

All the study districts highly significantly (p<0.001) suffered from starvation due milk loss during outbreaks which agree with [29] who reported that households and communities that depend more or solely on livestock for their livelihoods suffer more during FMD outbreaks since livestock provide a safety net for farmers throughout the developing world [30]. However, the current findings defer in that districts that were less dependent on livestock for their livelihoods suffered from starvation more than Nakaseke that had a higher sole dependency probably because Nakaseke had the lowest prevalence for FMD.

FMD outbreaks caused financial losses at the farmer and regional levels within inland and border districts as well as at the national level. FMD outbreaks caused even more impact on cattle sector performance due to losses incurred from reduced productivity, treatment, and control costs besides the trade bans [31]. In agreement with other studies, efficiency was undermined, and all actors in the cattle-beef marketing chain lost significant income streams during the outbreaks [32]. At the processing level, various actors lost income amounting to 77% which means that during the outbreaks the actors only earn 23%, which severely affects their livelihoods, food security, and ability to meet basic needs in their households [8,7].

FMD outbreaks affected farmers and beef marketing by lowering cattle and meat prices with a resultant loss of income which agrees with earlier studies [33,34]. Cattle traders were affected during the FMD outbreaks because quarantine period made it difficult for them to obtain stock from the affected areas and at the same time they faced difficulty in finding alternative suppliers thus they were rendered jobless. Consequently, it was difficult for the processors to obtain slaughter animals during the same period.

The government of Uganda should retain responsibility for controlling and preventing FMD since this disease affects the poor most who cannot afford to control or prevent the disease without government support. The magnitude of financial losses estimated justifies investments in prevention and control of FMD at all levels of the cattle marketing chain.

## Conclusion

All actors along the cattle marketing chain incur losses during FMD outbreaks, but smallholder farmers are most affected. Control and prevention of FMD should remain the responsibility of the government if Uganda is to achieve a disease-free status that is a prerequisite for free movement and operation of cattle markets throughout the year which will boost cattle marketing.

## Authors’ Contributions

The author was the principal investigator and she designed the study, prepared the data collection instruments and collected the data with the help of research assistants, analyzed the data and wrote the manuscript that was submitted for this publication.
